# RedeR: R/Bioconductor package for representing modular structures, nested networks and multiple levels of hierarchical associations

**DOI:** 10.1186/gb-2012-13-4-r29

**Published:** 2012-04-24

**Authors:** Mauro AA Castro, Xin Wang, Michael NC Fletcher, Kerstin B Meyer, Florian Markowetz

**Affiliations:** 1 Cancer Research UK Cambridge Research Institute and Department of Oncology, University of Cambridge, Robinson Way, Cambridge CB2 0RE, UK

## Abstract

Visualization and analysis of molecular networks are both central to systems biology. However, there still exists a large technological gap between them, especially when assessing multiple network levels or hierarchies. Here we present RedeR, an R/Bioconductor package combined with a Java core engine for representing modular networks. The functionality of RedeR is demonstrated in two different scenarios: hierarchical and modular organization in gene co-expression networks and nested structures in time-course gene expression subnetworks. Our results demonstrate RedeR as a new framework to deal with the multiple network levels that are inherent to complex biological systems. RedeR is available from http://bioconductor.org/packages/release/bioc/html/RedeR.html.

## Rationale

Biological networks contain modules of genes or proteins that may function in the same pathway [1]. As genes or proteins inside a module can be co-regulated, they are often represented by one single node in the network [2]. Such modules can be inferred by a number of statistical methods and the results are usually represented in graphs [3,4]. Given the complex associations that can take place in these graphs, it is a challenge to infer and visualize multiple levels or hierarchies within and between subnetwork structures.

Popular software like Cytoscape [5] provide a general framework to deal with part of this complexity by providing software plugins and visualizing networks in flat topologies. Flat networks are largely adequate to deal with different graph elements, as long as the network levels stay small. However, when describing and defining functional modules a hierarchical data structure is more appropriate because this enables the construction of graph elements within modules in a scalable system (for example, chains of nested networks). Herein we present RedeR, an R package combined with a Java core engine to cope with hierarchical and nested network structures.

RedeR is designed to deal with three key challenges in network analysis. Firstly, biological networks are modular and hierarchical, so network visualization needs to take advantage of such structural features to avoid cluttered and uninformative 'hairballs'. Secondly, network analysis relies on statistical methods, many of which are already available in resources like CRAN or Bioconductor. However, the missing link between advanced visualization and statistical computing makes it hard to take full advantage of R packages for network analysis. Thirdly, in larger networks user input is needed to focus the view of the network on the biologically relevant parts, rather than relying on an automatic layout function.

RedeR is designed to address these challenges: (i) we implement modular objects for subnetworks that allow to easily lay out and analyze network modules and their connections; (ii) the software is tightly integrated to R - while RedeR visualizes R outputs, its results can be directly fed back into R for further statistical analyses, which makes the power of R available for users primarily interested in visualization but not statistical computing; and (iii) we implement a dynamic layout that directly reflects user input.

We exemplify RedeR's visualization and analysis capabilities in a case study based on the re-analysis of gene expression and chromatin immunoprecipitation (ChIP)-on-chip data from an estrogen receptor (ER) study in the MCF-7 breast cancer cell line [6]. We anticipate that RedeR will be useful for integrative analyses and deriving gene expression networks that demand complex data abstraction and multiple network levels.

## Overview of the software

RedeR is distributed as an R/Bioconductor package. It is implemented by S4 classes in R [7] combined with Java graphical user interface. Standard Java Swing components and the NetBeans IDE 6.9 development environment [8] were extensively used to implement the graphical interface, which operates in conjunction with R libraries. In what follows we describe the implementation of the main features of the software.

### User-friendly interface in R

**Figure 1:**
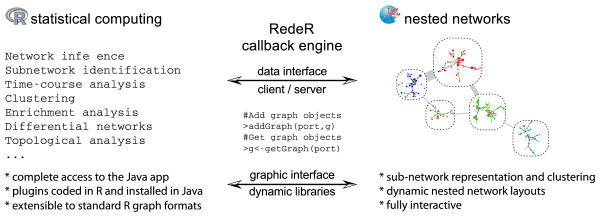
**Schematic representation of the RedeR callback engine**. In the low-level interface, the Apache xmlrpc webserver [9] is used to link R and Java.

The software uses different strategies to link R to Java (Figure 1). For the data interface, the callback engine makes calls from R via xml-rpc protocol by setting R as client and the Java app as server. For the graphic interface, the calls are executed from the Java core through dynamic libraries (that is, it wraps R graphics into Java classes). Four packages are essential to build the interface. At the Java side, the software uses the Apache xmlrpc webserver [9] and the JRI library interface [10]; at the R side, it uses the XMLRPC and rJava packages [11,12]. RedeR is invoked from R by the method 'calld':

# Set the server port and invoke the Java app

> library(RedeR)

> rdp <- RedPort()

> calld(rdp)

This method sets the environment and all paths required to start the callback engine, after which the software can either interact with R or run as a stand-alone application. The graphic interface is extensively controllable from the R command line and provides several menus that allow basic actions, such as selecting nodes and changing their appearance. In order to maintain a high level of compatibility, all methods in the R interface use *igraph *objects as prototype data format.

### Unique data structure for hierarchical networks

**Figure 2:**
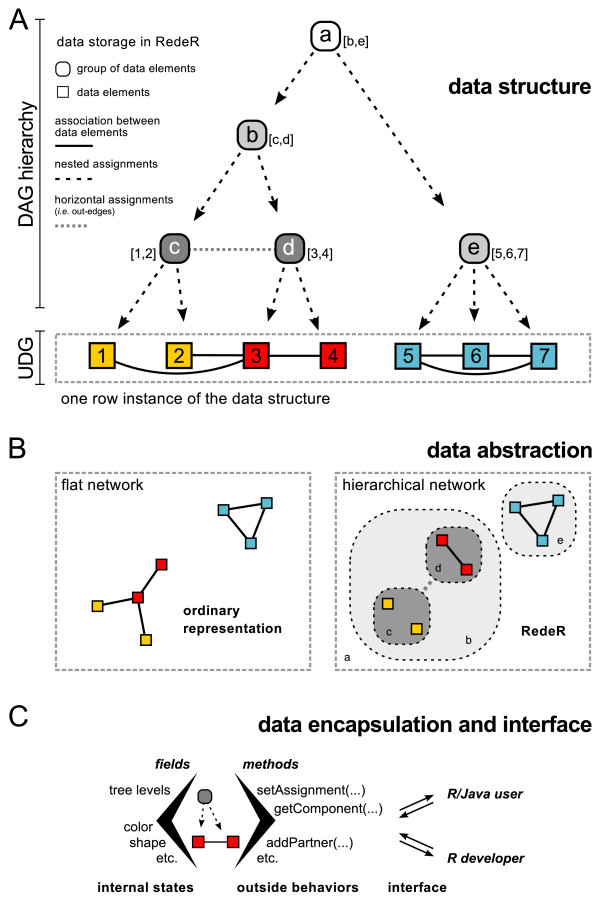
**Schematic representation of RedeR data packing and storing**. **(a) **Data structure. The software emulates a mixed graph with two layers and multiple levels in order to organize and manage the hierarchical associations. **(b) **Data abstraction. For the end-user, the data abstraction corresponds to the network layout that represents the data structure. A flat network is shown to contrast an ordinary representation (left) with RedeR hierarchical topology (right). **(c) **Data encapsulation: end-users and R developers have access to the outer level of the application through the methods handled by the interface.

The schematic representation of how data are stored in RedeR is depicted in Figure 2. The data structure emulates a mixed graph with two layers and multiple levels. The first layer can be defined by a directed acyclic graph (DAG) with no more than one parent for each vertex, essentially a data tree with multiple branches and levels, and with no cycles (Figure 2a). The second layer is designed as an undirected graph (UDG) on the lowest level of the DAG hierarchy. Horizontal or non-hierarchical associations can also be reassigned from one layer to another, external to the hierarchical structure. This data organization is depicted in Figure 2b and corresponds to the topology that could represent a given dataset (for example, protein-protein interaction networks, ontologies, and so on). Flat networks, as illustrated in the left graph, can represent just one row instance of the data structure (that is, not divided into modules or layers). In contrast, hierarchical networks, as illustrated in the right graph, support a modular organization and can exhibit the complete information. The design of the software encapsulates the data structure within subclasses of a graph blueprint that contains all fields and methods common to both data types (Figure 2c). This design is implemented in the Java core and is extended to R to provide users and developers full access to the outer level of the application. As an initial demonstration of the software, next we provide a chunk of R that generates modular structures as illustrated in Figure 2b.

# Generate an igraph object (a toy example with modular structures)

> g <- gtoy.rm(m = 5, nmax = 30)

# Compute a hierarchical clustering using standard R functions

> hc<-hclust(dist(get.adjacency(g)))

# Add graph to RedeR

> addGraph(rdp, g)

> nesthc(rdp, hc, metric="rootdist", cutlevel = 3, nlev = 1)

This toy example maps one level of the hierarchy onto the network topology. Additional levels and different sections of the hierarchy can be mapped using the same function (for further details, please see 'nesthc' documentation in the R package).

### Dynamic layout modeling

One of the most versatile features of the software is the ability to deal with nested network objects using dynamic modeling, which makes it possible to represent, for example, subnetworks and time-series onto the same graph in a user-friendly routine. The layout uses force-directed algorithms as described elsewhere [13,14]. Here we adapted the method to deal with nested networks. In force-directed graphs, each edge can be regarded as a spring - with a given target length - and can either exert a repulsive or attractive force on the connected nodes, while nodes are analogous to mutually repulsive charged particles that move according to the applied forces. In RedeR, the layout is additionally constrained by the hierarchical structure. For example, a nested node is constrained to its parent node by opposing forces applied by the nest, which is regarded as a special node whose nested objects can reach a local equilibrium independently from other network levels. The layout is adjusted by global options and evolves iteratively (and interactively) until the system reaches the equilibrium state. It can be started via either the graphical user interface or the R command line, as for example:

# Start dynamic layout

> relax(rdp, ps=TRUE)

# Reset graph

> resetd(rdp)

The user can observe all steps of the layout optimization process and, at any particular time, the process can be driven interactively. In this sense, 'dynamic' not only refers to the iteration steps required to layout a graph by the force-direct algorithm but also is related to the user's interaction. This option is particularly useful for additional control over containers and nested nodes in hierarchical structures. We also added to the Java core some popular static layout algorithms from open source libraries [15] as a complementary option to the list of all static layouts that can either be found in the R package collections or, as usual, customized in R by the user.

### R code deployment

R developers can deploy R code to RedeR by using the 'PluginBuilder' method. This feature provides a direct way to extend existing R packages to the Java interface. The combination of R and Java code in a mark-up construct gives rise to this extensible feature. The idea is based on the successful framework used by the Sweave package that mixes LaTeX syntax and R codes in order to parse R text chunks within LaTeX documents. In RedeR, the plugins are exported to the Java core by the 'submitPlugin' function. On the other side of the interface, the software receives the request, stores the new method in an XML document and mounts the plugin in the application, including submenus in the main panel. RedeR plugins have two main sections: methods and add-ons. The 'methods' section can be regarded as the plugin trigger. When installed in the Java app, this trigger starts a given analysis by unfolding R expressions wrapped in the methods. Add-ons use the same strategy, but remain hidden in the app and can either load formal functions or pass additional arguments to R (a code sample is provided in RedeR vignette, *plugin builder *tutorial).

### Pre-processed data and documentation

The pre-processed data used in the case study were obtained by the LIMMA package [16]. An R script that reproduces the analysis is available in the supplements. Additionally, the R package provides extensive documentation for all methods available in the software, including description of the data objects, examples, and a tutorial introducing the main functionalities.

## Case study

In this section we demonstrate some essential features of the software in two examples based on the re-analysis of ChIP-on-chip and gene expression data from a genome-wide study describing ER binding sites in the MCF-7 breast cancer cell line [6]. The ChIP-on-chip dataset consists of a Bed file containing the genome position of 3,665 unique ER binding sites, while the gene expression data consist of 12 time-course Affymetrix U133Plus2.0 microarrays from MCF-7 cells stimulated with estrogen for 0, 3, 6 and 12 h (all arrays in triplicate).

### Biological background

The purpose of the study by Carroll *et al*. [6] is the identification of new authentic *cis *ER binding sites and ER target genes in breast cancer cells. One of the challenges faced by the authors was that only a small fraction (4%) of the ER binding sites mapped to promoter-proximal regions, within 1 kb of the transcription start sites. More frequently, ER binding sites are found at considerable distance from the regulated gene and only one-third of early estrogen up-regulated genes contain ER binding sites within 50 kb of the transcription start site. This finding has made it difficult to validate ER-regulated candidate genes as there may be multiple genes within the 100 kb interval of the ER binding site and because the usual association of transcription factor binding sites and promoter regions occurs in only a minority of cases.

### Hierarchical and modular organization in gene co-expression networks

**Figure 3:**
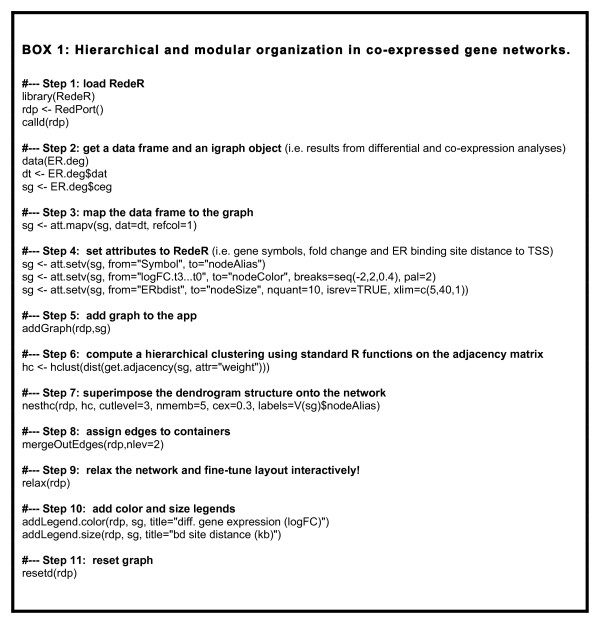
**Hierarchical and modular organization in co-expressed gene networks**. R script describing step-by-step all intermediate R objects required to obtain the results presented in the first case study. TSS, transcriptional start site.

The aim of this example is to examine the hierarchical structure of co-expressed gene network modules. A step-by-step description of the analysis is provided in Figure 3. Three standard R objects are used: a data frame object with many gene attributes inferred from the Carroll *et al*. [6] dataset (for example, differentially expressed genes, log fold change values and ER binding site distance), the corresponding gene expression matrix and an igraph object obtained by co-expression analysis on genes differentially expressed at 3 h (further details on the pre-processed data can be found in Additional file 1).

**Figure 4:**
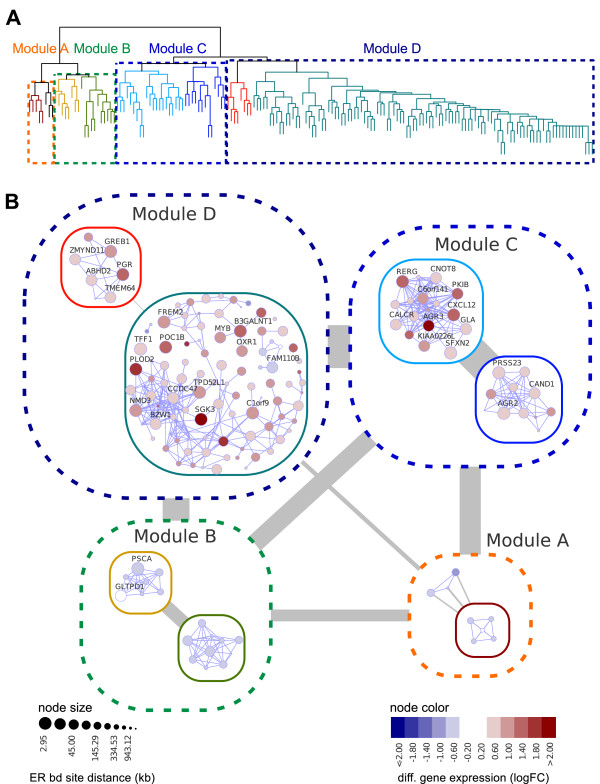
**Hierarchical networks**. **(a) **Dendrogram derived from complete-linkage clustering analysis using Euclidean distance on the gene expression matrix of all genes differently expressed at 3 h (related to 0 h) in estrogen-treated MCF-7 cells (Carroll *et al*. [6] dataset). **(b) **Hierarchical network obtained by superimposing the dendrogram onto the corresponding co-expression gene network. The co-expression associations were computed for the same set of genes (for additional details on the pre-processed data please see Additional file 1 and Figure 3). Node coloring depicts differential expression as log2 fold-change (logFC) and node size indicates the kilobase distance of the transcription start site to the nearest ER binding site. Out-edge width represents the sum of all edges between modules divided by the total possible edges.

Figure 4a shows a hierarchical clustering obtained on the adjacency matrix and in Figure 4b we display two levels of such hierarchical organization onto the co-expression gene network. For each gene its size indicates the proximity to the nearest ER binding site (large nodes correspond to genes close to the ER binding sites) and the colors represent the log2 differential expression values.

Taken together, this case study not only illustrates how to constrain the network topology by a hierarchical structure, but also raises an interesting biological observation. The identification of co-regulated gene modules is one of the key steps towards understanding genetic regulatory networks. However, similar patterns in gene expression modules are not directly associated with a common mechanism of gene regulation. The identification of co-regulated modules is far from trivial and this case study provides a simple workflow to inspect in detail potentially co-expressed gene modules that share binding sites for the same transcription factor. The software permits visualizing these individual gene modules, displaying each individual component and the connections between them, as well as the hierarchical associations between modules and genes.

### Nested structures in time-course gene expression subnetworks

**Figure 5:**
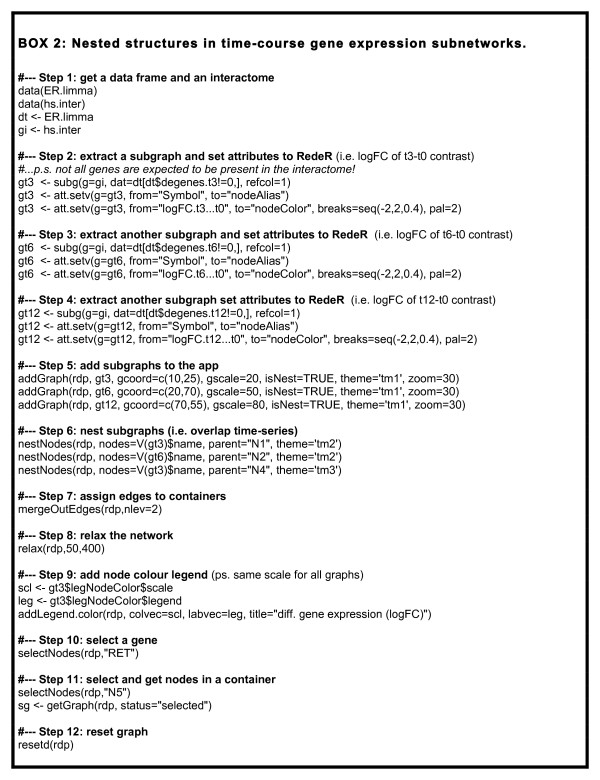
**Nested structures in time-course gene expression subnetworks**. R script describing step-by-step all intermediate R objects required to obtain the results presented in the second case study. logFC, log2 fold-change.

Another common approach to analyze complex biological datasets is the use of prior knowledge. Using the HPRD database [17] as prior information, in Figure 5 we describe a workflow that maps to the human interactome all differentially expressed genes inferred in estrogen-treated MCF-7 cells (that is, genes differentially expressed at 3, 6 and 12 h related to 0 h), and for each time point we select the largest subnetwork in order to demonstrate how RedeR represents nested structures.

**Figure 6:**
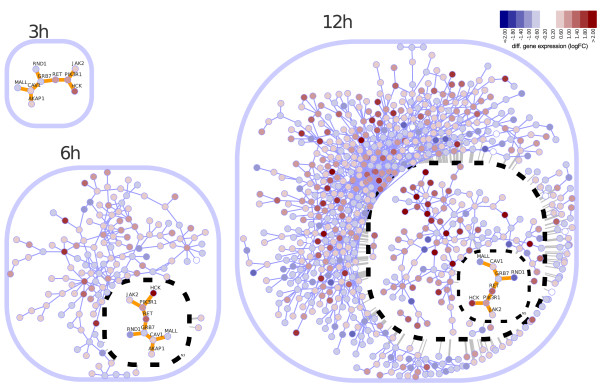
**Nested subnetworks**. Genes differentially expressed in estrogen-treated MCF-7 cells at 3, 6 or 12 h (relative to 0 h; Carroll *et al*. [6] dataset) were mapped to the human interactome (HPRD database [17]) and for each time point the largest subnetwork was selected in order to demonstrate how RedeR represents nested structures (additional details in Figure 5). Node coloring depicts differential expression as log2 fold-change (logFC). The insets correspond to the overlap between consecutive time points.

Accordingly, among the possible outcomes one may expect to see gene modules that are (i) induced early after stimulus, (ii) continuously stimulated/repressed, or (iii) respond later to the treatment. Given the partial- or non-overlapped time-course responses and the different module sizes, such a scenario can give rise to a complex data structure. Figure 6 shows these modules as a chain of nested subnetworks. Two observations are evident from this comparison across time series: as time goes by the subnetwork gets bigger but the core remains nearly the same. Such abstracted structure shows that the early response differentially expressed subnetworks are nested to the subsequent and larger gene modules, suggesting a stepwise and progressive gene expression response in estrogen-treated MCF-7 breast cancer cells. Additional file 1 provides supporting material to extend this case study to more advanced scenarios further illustrating the type of questions that can be explored by using RedeR.

## Benchmark

### RedeR in the context of gold-standard software in the same field

There are other excellent tools to represent and analyze biological networks, and each has their own unique features. Table 1 describes RedeR in the context of gold standard software in the same field. In R three packages provide standard software infrastructures to deal with graphs: igraph, graph and Rgraphviz [18-21]. However, the rendering process of these packages relies on R's internal plotting libraries, which lack interactive capabilities. Some simple interactive options are available in igraph, but according to the authors these features are not very well developed at this stage. In contrast, RedeR provides a robust graph-handling engine that is directly extensible to R objects; therefore, those used to work with graphs in R can easily become familiar with RedeR.

**Table 1:** RedeR in the context of gold-standard network visualization software and R

	RedeR	Cytoscape	Graphviz	igraph	graph
**Software design**					
Hierarchical data structure^a^	Yes**^b^**	No	Yes**^b^**	No	No
Data abstraction^a^	Modular**^b^**	Flat	Layered**^b^**	Flat	Flat
Data encapsulation^a^	Yes	Yes	No	No	No
Core engine	Java	Java	DOT	C	R
R interface	R <-> Java	R -> Java	R <- DOT	R <- C	Pure R
Deployment to R	Embedded	External	External	Embedded	Embedded
Plugin coding language	R	Java	DOT language**^c^**	C**^d^**	-
					
**Selected features**					
Scalability on nested networks^e^	Yes	No**^f^**	Yes	No**^g^**	No**^g^**
Interactive graph handling	Yes	Yes	No	Partially**^h^**	No
Dynamic layouts	Yes**^i^**	No	No	No	No
Comparison across multiple nested networks	Yes	No	No	No	No
Panels^j^	Yes	No	No	No	No

**^a^**For further definitions, please see the Implementation section and Figure 2. **^b^**In RedeR, the hierarchy is encapsulated, so users deal with methods. In Graphviz users implement methods by themselves in DOT language. This difference has an important effect on the ability of the software to deal with modular structures, such as recycling data objects used in subnetworks. **^c^**The plugin infrastructure is available for Graphviz. **^d^**Provides a C library to be used in third-party applications. **^e^**Able to accommodate increasing amounts of nested objects. **^f^**In Cytoscape, nested networks are represented as images inside nodes. For each nested network one image is required. Images are not scalable, so the hierarchy cannot be extended to other levels. **^g^**In these applications, modules can be represented by layering images, which is essentially a drawing process. **^h^**According to the authors, the interactive features are not very well developed at this stage. **^i^**In RedeR, the dynamic layout is also extensible to the nested structures. **^j^**This feature provides support for multiple panels in the same graph, representing subnetworks, and so on.

Another option is the package RCytoscape [22]. This R package implements via CytoscapeRPC [23] an interface to Cytoscape [5], which can be regarded as a gold standard software for network visualization. Although robust and easy to use, Cytoscape is designed mainly to deal with flat network topology, which does not accommodate increasing amounts of nested objects. For example, using flat topology to represent a chain of nested networks, the number of graphs would increase proportionally with the network levels. Using RedeR, the job can be performed in just one graph (Figure 2b, data structure section). In this sense, RedeR constitutes a new option to assess networks with multiple levels or hierarchies, and this is a surprisingly common situation in biological networks.

### Performance

**Figure 7:**
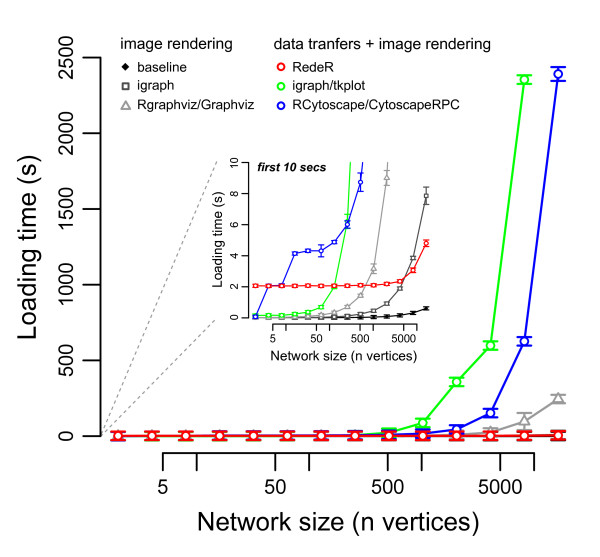
**Performance of six graph tools loading scale free networks of increasing size**. Each point in the plot corresponds to the average elapsed time (in seconds) required to load one of these networks. The inset shows the first 10 s of the same tests (± standard deviation, *n *= 10). The networks were obtained by the function 'barabasi.game' available in the R package igraph [19]. Briefly, each network has ϖ vertices and ε edges generated by a step-model where the first step generates a single vertex and no edge; the subsequent steps generate one vertex linked to an old vertex according to a probability distribution proportional to the degree of the nodes; therefore, in the end there are ϖ-1 edges. The networks were obtained and set prior to the performance tests, and only with minimum information in order to guarantee equal conditions among the software (that is, without any graph attribute, such as color, size, and so on). Versions tested: R version 2.14.1, RedeR_1.0.3, igraph_0.5.5-4, RCytoscape_1.4.3, Cytoscape 2.8.1, CytoscapeRPC 1.7, Rgraphviz_1.32.0. Platform: x86_64-apple-darwin9.8.0/x86_64 (64-bit). Hardware: MacPro4.1 2 × Quad-core Intel Xeon 2.26 GHz, 6 GB RAM. The source code used to run the complete analysis is available in Additional files 2 and 3.

In order to benchmark shared functionalities among these packages, we tested the performance for loading scale-free networks of increasing size, up to the human interactome scale. The results are shown in Figure 7 and the R script used to run the complete analysis is available in Additional files 2 and 3. Although any benchmark is restricted [24], it is clear from these results that RedeR performs very well, even compared to packages that only deal with image rendering. One remarkable aspect is that RedeR maintains its level of performance when tested by larger networks, only comparable to the baseline, which is a simple test to assess the speed of R for plotting dots and lines (that is, image rendering). The software continues to track the baseline, but transfers network information at the same time (Figure 7, inset). Another remarkable aspect is observed at the maximum loading time. For example, while RedeR took 4.8 s (± 0.2 s) to load a network with 16,384 nodes, RCytoscape/CytoscapeRPC required 2,391.3 s (± 45.7 s) to load the same graph (further details in the legend of Figure 7 and Additional files 2 and 3).

## Conclusions

In this work we introduced RedeR, a software designed for the representation of nested and hierarchical biological networks. The ability to perform advanced visualization tightly integrated to R allows RedeR to take full advantage of R packages for network analysis and statistical computing. Likewise, RedeR is an ongoing project that provides a comprehensive and entirely new framework to read, write and manipulate R code mixed to a Java data structure. Its architecture allows the creation of R-based plugins with minimum effort, potentially extending the existing R packages to different communities of users interested in studying biological networks.

Rather than analyzing a single network, current research focuses on differences in networks, re-wiring events, as well as higher-level, modular characteristics of networks. These can be hard to visualize in standard tools. RedeR implements a framework for network comparison and module representation by introducing a hierarchy of 'containers' in which many networks and their connections can be visualized at the same time. We anticipate that our software will be particularly useful to assess datasets that demand detailed analysis of inter- and intra-modular associations.

## System requirements

R (version>=2.14) and Java Runtime Environment (version>=5). Available since Bioconductor 2.9.

## Abbreviations

ChIP: chromatin immunoprecipitation; DAG: directed acyclic graph; ER: estrogen receptor.

## Competing interests

The authors declare that they have no competing interests.

## Authors' contributions

Conceived the project: MAAC KBM FM. Supervised the project: KBM FM; Conceived and designed the software: MAAC XW FM. Implemented the software: MAAC. Implemented the analysis pipeline: MAAC XW. Conceived the case studies: MAAC XW MF KBM FM. Wrote the paper: MAAC KBM FM. All authors read and approved the final manuscript.

## Supplementary Material

Additional file 1 **Pre-processed data analysis**. PDF document describing the pre-processed data analysis, including three examples illustrating how RedeR can be integrated with other R packages. Click here for file

Additional file 2 **Benchmark source code**. R script used to run the benchmark analysis. Click here for file

Additional file 3 **Complementary benchmark source code**. R script with complementary R functions required in the benchmark. Click here for file
