# Supplementary material for: RedeR: R/Bioconductor package for representing modular structures, nested networks and multiple levels of hierarchical associations
Source: Genome Biol. 2012 Apr 24;13(4):R29. doi: 10.1186/gb-2012-13-4-r29 (PMC3446303; doi:10.1186/gb-2012-13-4-r29)
Supplement: Additional file 1 — Pre-processed data analysis. PDF document describing the pre-processed data analysis, including three examples illustrating how RedeR can be integrated with other R packages. [file gb-2012-13-4-r29-S1.PDF]

Supporting Material

RedeR: an R/Bioconductor package for representing modular structures, nested networks and multiple levels of hierarchical associations

Mauro A. A. Castro, Xin Wang, Michael N. C. Fletcher, Kerstin B. Meyer, Florian Markowetz*

Cancer Research UK Cambridge Research Institute and Department of Oncology, University of Cambridge, Robinson Way, Cambridge CB2 0RE, UK.

Contact: florian.markowetz@cancer.org.uk

Table of Contents

Datasets **2**
Gene expression data *2*
ChIP-on-chip data *2*

Pre-processed data analysis **2**
Differential gene expression analysis *2*
Co-expression gene network analysis *3*
R data objects included in RedeR *3*

Additional examples **4**
Integration with Pvclust package *4*
Integration with BioNet package *5*
Integration with HTSanalyzeR package *6*

References **7**

Datasets

Gene expression data

The gene expression dataset consists of 12 time-course Affymetrix U133Plus2.0 microarrays: 3 replicates at 0h, 3 replicates at 3h, 3 replicates at 6h and 3 replicates at 12h. The original dataset is available at GEO database (GSE11324) [1].

ChIP-on-chip data

The gene estrogen receptor (ER) binding site dataset consists of a Bed file of ER ChIP-on-chip experiment [1]. The original dataset is available at http://research.dfci.harvard.edu/brownlab/datasets/index.php (ER sites from the Bed file '1E-5.bed').

Pre-processed data analysis

Differential gene expression analysis

The differential expression analysis was carried out in R using the LIMMA package [2]. The contrasts were set to obtain genes differentially expressed at 3, 6 and 12 h (related to 0 h). Additionally, the distance of the transcript start site (TSS) to the closest ER binding site is mapped to each gene and the results are combined in one single data frame called *ER.limma* available in the RedeR package. We provide next a step-by-step script to run the complete analysis:

```
#--- Step 1: load RedeR and limma packages
library(RedeR)
library(limma)
library(biomaRt)

#--- Step 2: load Carroll2006 datase
data(Carroll2006)

#--- Step 3: build design matrix
t <- factor(Carroll2006$tgs$Time)
design <- model.matrix(~0+t)

#--- Step 4: fit lm model
fit <- lmFit(Carroll2006$exp,design)
fit$genes$RefSeq <- Carroll2006$ids$RefSeqID
fit$genes$Symbol <- Carroll2006$ids$GeneSymbol

#--- Step 5: set contrasts
contrasts <- makeContrasts(t3-t0, t6-t0, t12-t0, levels=design)

#--- Step 6: eBayes correction and decision
ct.fit <- eBayes(contrasts.fit(fit, contrasts))
res.fit<-decideTests(ct.fit,method="global", adjust.method="BH", p.value=0.0001)

#--- Step 7: combine results from limma in one single data.frame
ER.limma <- data.frame( ENTREZ  = ct.fit$genes$ID, Symbol  = ct.fit$genes$Symbol,
            logFC = ct.fit$coef, p.value = ct.fit$p.value, degenes = unclass(res.fit), stringsAsFactors = FALSE)

#--- Step 8: get ER binding sites from Carroll2006 dataset
bdsites <- Carroll2006$bdsites

#--- Step 9: get TSS from biomaRt
# p.s. make sure to access GRCh37 build ( e.g. check " listDatasets(useMart("ensembl")) " )
mart <- useDataset("hsapiens_gene_ensembl", useMart("ensembl"))
tssmap <- getBM(attributes=c("entrezgene","chromosome_name","transcript_start"),
            filters="entrezgene", values=ER.limma$ENTREZ, mart=mart)
tssmap <- data.frame(tssmap, chrom=paste("chr", tssmap$chromosome_name,sep=""), stringsAsFactors=FALSE)

#--- Step 10: find the closest ER binding site for each TSS
delta_bd <- array(NA,dim=nrow(tssmap))
```

```
names(delta_bd) <- tssmap$entrezgene
for(i in 1:nrow(tssmap)){
        chrom    <- as.character(tssmap$chrom[[i]])
        txstart  <- tssmap$transcript_start[i]
        absdelta <- abs(txstart-bdsites$bdMean)
        absdelta[bdsites$bdChrom!=chrom] <- NA
        delta_bd[i] <- absdelta[sort.list(absdelta)[1]]
}

#--- Step 11: combine data frames
delta_bd <- data.frame(ENTREZ=names(delta_bd), bdDist=round(delta_bd/1000,2), stringsAsFactors=FALSE)
delta_bd <- aggregate(bdDist ~ ENTREZ, data=delta_bd, min)
rownames(delta_bd) <- delta_bd$ENTREZ

#--- Step 12: get the final data object
ER.limma <- data.frame(ER.limma, ERbdist=delta_bd[ER.limma$ENTREZ,"bdDist"], stringsAsFactors=FALSE)
```

Co-expression gene network analysis

The co-expression analysis was computed for early response genes (*i.e.* genes differentially expressed at 3 h) by the function *cea* available in RedeR. This function infers gene co-expression networks by computing a null distribution for correlation coefficients via permutation analysis and returns an adjacency matrix with significant correlation values. Additional detains are available in the R package documentation (*e.g.* help(cea) in R). The script described below reproduces the pre-processed data derived from the ER.limma data object and generates the ER.deg data object included in the R package (see next section: *R data objects included in RedeR*).

```
#--- Step 1: load RedeR
library(RedeR)

#--- Step 2: load data objects
data(Carroll2006)
data(ER.limma)

#--- Step 3: get all differentially expressed genes from the ER.limma data object (see Suppl. Box 1)
idx <- rowSums(ER.limma[,c(9,10,11)]!=0)
dat <- ER.limma[idx>0,]

#--- Step 4: get the gene expression matrix for the same set of genes
exp <- Carroll2006$exp[is.element(rownames(Carroll2006$exp), dat$ENTREZ),]

#--- Step 5: compute a co-expression gene network for genes differentially expressed at 3h (i.e. early response)
dat3 <- dat[dat$degenes.t3!=0, "ENTREZ"]
res <- cea(exp[dat3,], sig=1e-4, nper=1000, plotcea=FALSE, ptype=4)
ceg <- graph.adjacency(res, diag=FALSE, mode="undirected", weighted=TRUE)

#--- Step 6: get the largest connected component
ceg <- subg(g=ceg, dat=dat3, transdat=FALSE)

#--- Step 7: generate the same RData object included in the package
ER.deg <- list(dat=dat, exp=exp, ceg=ceg)
```

R data objects included in RedeR

1. _*Carroll2006*_: data frames with log2 gene expression data, microarray details, gene ids and ER binding sites mapped to genome build GRCh37 (see section *DATA USED IN THE CASE STUDY*).
2. _*hs.inter*_: igraph object with the Human interactome extracted from the Human Protein Reference Database (HPRD) in April 2011 ('name' attribute is mapped to ENTREZ ID).
3. _*ER.limma*_: data-frame containing pre-processed results from limma analysis and ER binding sites mapped to differentially expressed (DE) genes. Content: annotation (ENTREZ and Symbol), time-course fold change (logFC.t3, logFC.t6, logFC.t12), p values (p.value.t3, p.value.t6, p.value.t12), DE genes (degenes.t3, degenes.t6, degenes.t12) and distance of the closest ER bd site to the TSS – in kb (ERbdist).

4. *ER.deg*: data-frame containing: summary of ER.limma data object with extracted data for differentially expressed genes only; corresponding data matrix with log2 gene expression values; and co-expression gene network of early ER-responsive genes computed by the function “cea”.

Additional examples

This section presents three examples to illustrate how RedeR can be integrated with other packages in R.

Integration with Pvclust package

This example is similar to the first one discussed in the paper (see case study and Fig.3 in the paper). Here the analysis is extended to derive clusters on statistical bases by the package Pvclust [3], which computes p-values for the hierarchical clustering via multiscale bootstrap resampling. The output from the analysis consists of an object of class *pvclust*, which is sent to RedeR in order to map significant modules onto a graph.

```
#--- Step 1: load RedeR and Pvclust packages
library(RedeR)
library(pvclust)

#--- Step 2: start the interface
rdp<-RedPort()
calld(rdp)

#--- Step 3: get a data frame, gene expression matrix and an igraph object
data(ER.deg)
dt <- ER.deg$dat
sg <- ER.deg$ceg
gx <- ER.deg$exp

#--- Step 4: map the data frame to the graph
sg <- att.mapv(sg, dat=dt, refcol=1)

#--- Step 5: set attributes to RedeR (i.e. gene symbols, fold change and ER binding site distance to TSS)
sg <- att.setv(sg, from="Symbol", to="nodeAlias")
sg <- att.setv(sg, from="logFC.t3...t0", to="nodeColor", breaks=seq(-2,2,0.4), pal=2)
sg <- att.setv(sg, from="ERbdist", to="nodeSize", nquant=10, isrev=TRUE, xlim=c(5,40,1))

#--- Step 6: add graph to the app
addGraph(rdp,sg)

#--- Step 7: compute a hierarchical clustering using Pvclust

#...compute clustering on the gene expression matrix
#...set nboot=1000 or larger; pvclust parallel computing might be required!
gx <- gx[V(sg)$name,]
hc <- pvclust(t(gx), method.hclust="average", method.dist="euclidean", nboot=100)

#...check results (e.g. significant clusters)
plot(hc)

#--- Step 8: superimpose significant gene modules onto the network as mapped in clustering analysis
nesthc(rdp,hc, nmemb=5, cex=0.3, labels=V(sg)$nodeAlias)

#--- Step 9: assign edges to containers
mergeOutEdges(rdp)

#--- Step 10: relax the network (p.s. fine-tune layout and container size interactively!)
relax(rdp)

#--- Step 11: add color and size legends
addLegend.color(rdp, sg, title="diff. gene expression (logFC)")
addLegend.size(rdp, sg, title="bd site distance (kb)")

#--- reset graph
resetd(rdp)
```

Integration with BioNet package

Similarly to the second example discussed in the paper (see case study and Fig.4), the next workflow describes how to obtain optimal subnetworks using the R package BioNet [4]. This package provides tools to identify differentially expressed subnetworks within large *a priori* defined networks (*e.g.* the human interactome). The output from BioNet consists of *igraph* objects, which are then used to demonstrate nested structures.

```
#--- Step 1: load RedeR and BioNet packages
library(RedeR)
library(BioNet)

#--- Step 2: start the interface
rdp<-RedPort()
calld(rdp)

#--- Step 3: get a data frame with pvals from limma analysis and an interactome
data(ER.limma)
data(hs.inter)
dt <- ER.limma
gi <- hs.inter

#--- Step 4: map the data frame to the interactome (p.s. not all genes are present in the interactome!)
subnet <- subg(g=gi, dat=dt, refcol=1)
subnet <- att.setv(g= subnet, from="Symbol", to="nodeAlias")

#--- Step 5: run BioNet for each time point (using pvals from limma) and get optimal scoring modules

#...get optimal scoring module for t3-t0 contrast using BioNet
pvals <- dt$p.value.t3
names(pvals) <- dt$ENTREZ
fb <- fitBumModel(pvals, plot=FALSE)
scores <- scoreNodes(network=subnet, fb=fb, fdr=1e-05)
gt3 <- runFastHeinz(network=subnet, scores=scores)

#...get optimal scoring module for t6-t0 contrast using BioNet
pvals <- dt$p.value.t6
names(pvals) <- dt$ENTREZ
fb <- fitBumModel(pvals, plot=FALSE)
scores <- scoreNodes(network=subnet, fb=fb, fdr=1e-05)
gt6 <- runFastHeinz(network=subnet, scores=scores)

#...get optimal scoring module for for t12-t0 contrast using BioNet
pvals <- dt$p.value.t12
names(pvals) <- dt$ENTREZ
fb <- fitBumModel(pvals, plot=FALSE)
scores <- scoreNodes(network=subnet, fb=fb, fdr=1e-05)
gt12 <- runFastHeinz(network=subnet, scores=scores)

#--- Step 6: set RedeR attributes for each module (i.e. logFC for tx-t0 contrasts)
gt3  <- att.setv(g=gt3, from="logFC.t3...t0", to="nodeColor", breaks=seq(-2,2,0.4), pal=2)
gt6  <- att.setv(g=gt6, from="logFC.t6...t0", to="nodeColor", breaks=seq(-2,2,0.4), pal=2)
gt12 <- att.setv(g=gt12, from="logFC.t12...t0", to="nodeColor", breaks=seq(-2,2,0.4), pal=2)

#--- Step 7: add time-series to the app
n0 <- addGraph(rdp, gt3, gcoord=c(10,25), gscale=20, isNest=TRUE, theme='tm1', zoom=30)
n1 <- addGraph(rdp, gt6, gcoord=c(20,70), gscale=50, isNest=TRUE, theme='tm1', zoom=30)
n2 <- addGraph(rdp, gt12, gcoord=c(70,55), gscale=80, isNest=TRUE, theme='tm1', zoom=30)

#--- Step 8: nest subgraphs (i.e. overlap time-series)
nestNodes(rdp, nodes=V(gt3)$name, parent="N1", theme='tm2')
nestNodes(rdp, nodes=V(gt6)$name, parent="N2", theme='tm2')
nestNodes(rdp, nodes=V(gt3)$name, parent="N4", theme='tm3')

#--- Step 9: assign edges to containers and relax the network
mergeOutEdges(rdp,nlev=2)
relax(rdp,50,400)

#--- Step 10: add node color legend (ps. same legend is available in all graphs)
addLegend.color(rdp, colvec=gt3, title="diff. gene expression (logFC)")

#--- reset graph
resetd(rdp)
```

Integration with HTSanalyzeR package

The package *HTSanalyzeR* [5] uses an *a priori* established gene set collection to add interpretability to high-dimensional data. In this example the Gene Ontology (GO) database [6] is used to assess differentially enriched gene sets in the time-course expression data. Two complementary statistics are computed: a hypergeometric test and GSEA enrichment scores. For both cases a network representation is used to assess the overlap among the significant GO terms.

```
#--- Step 1: load RedeR and HTSanalyzeR packages -- and the gene set collections
library(RedeR)
library(HTSanalyzeR)

#--- Step 2: load gene set collections and snow for parallel processing
library(GO.db)
library(org.Hs.eg.db)
library(snow)

#--- Step 3: start the interface
rdp<-RedPort()
calld(rdp)

#--- Step 4: get a data frame from limma analysis and extract early differentially expressed genes (3 h)
data(ER.limma)
pheno <- ER.limma$logFC.t3
names(pheno) <- ER.limma$ENTREZ
hits <- ER.limma[ER.limma$degenes.t3!=0, "ENTREZ"]

#--- Step 5: get gene set collections from GO db
gos<-c("BP","MF","CC")
gsca<-list()
for(i in gos) gsca[[i]] <- GOGeneSets(species="Hs", ontologies=i)

#--- Step 6: run HTSanalyzeR using as 'hits' DE genes from limma analysis

#...create an object of class 'GSCA'
gsca <- new("GSCA", listOfGeneSetCollections=gsca, geneList=pheno, hits=hits)

#...do preprocessing
gsca <- preprocess(gsca, species="Hs", initialIDs="Entrez.gene")

#...do hypergeometric tests and GSEA using parallel processing
#...p.s.1:snow package might require customize settings
#...p.s.2:set nPermutations=1000 or larger
options(cluster=makeCluster(4, "SOCK"))
gsca <- analyze(gsca, para=list(pValueCutoff=0.05, nPermutations=100, minGeneSetSize=100))
stopCluster(getOption("cluster"))

#...append GO Terms
gsca <- appendGSTerms(gsca, goGSCs=gos)

#...get results (i.e. enrichment maps)
ghype<-list()
for(i in gos) ghype[[i]] <- viewEnrichMap(gsca, resultName="HyperGeo.results", gscs=i, gsNameType="term", plot=FALSE)
ggsea<-list()
for(i in gos) ggsea[[i]] <- viewEnrichMap(gsca, resultName="GSEA.results", gscs=i, gsNameType="term", plot=FALSE)

#--- Step 7: set a theme for common attributes
mytheme <- list(isNest=TRUE, gscale=35, nestFontSize=30, zoom=50, theme=1)

#--- Step 8: add HyperGeo results
labs <- paste("HyperGeo", gos)
for(i in 1:3) addGraph(rdp, ghype[[i]], theme=c(mytheme,nestAlias=labs[i]), gcoord=c(25*i,30))

#--- Step 9: add GSEA results
labs <- paste("GSEA", gos)
for(i in 1:3) addGraph(rdp, ggsea[[i]], theme=c(mytheme,nestAlias=labs[i]), gcoord=c(25*i,70))

#--- reset graph
resetd(rdp)
```

References

1. Carroll JS, Meyer CA, Song J, Li W, Geistlinger TR, Eeckhoute J, Brodsky AS, Keeton EK, Fertuck KC, Hall GF *et al*: **Genome-wide analysis of estrogen receptor binding sites**. *Nat Genet* 2006, **38**(11):1289-1297.

2. Smyth GK: **Linear models and empirical bayes methods for assessing differential expression in microarray experiments**. *Stat Appl Genet Mol Biol* 2004, **3**:Article3.

3. Suzuki R, Shimodaira H: **Pvclust: an R package for assessing the uncertainty in hierarchical clustering**. *Bioinformatics* 2006, **22**(12):1540-1542.

4. Beisser D, Klau GW, Dandekar T, Muller T, Dittrich MT: **BioNet: an R-Package for the functional analysis of biological networks**. *Bioinformatics* 2010, **26**(8):1129-1130.

5. Wang X, Terfve C, Rose JC, Markowetz F: **HTSanalyzeR: an R/Bioconductor package for integrated network analysis of high-throughput screens**. *Bioinformatics* 2011, **27**(6):879-880.

6. Ashburner M, Ball CA, Blake JA, Botstein D, Butler H, Cherry JM, Davis AP, Dolinski K, Dwight SS, Eppig JT *et al*: **Gene ontology: tool for the unification of biology. The Gene Ontology Consortium**. *Nat Genet* 2000, **25**(1):25-29.
